# Resorption Rates of Bone Graft Materials after Crestal Maxillary Sinus Floor Elevation and Its Influencing Factors

**DOI:** 10.3390/jfb15050133

**Published:** 2024-05-17

**Authors:** Ling Jing, Baohui Su

**Affiliations:** College of Biomedical Engineering, Sichuan University, Chengdu 610065, China; lychoa@outlook.com

**Keywords:** sinus floor augmentation, bone transplantation, bone resorption, CBCT

## Abstract

The aim of this study is to analyze the resorption rate of bone graft materials after crestal sinus floor elevation, study its influencing factors, and improve the long-term success rate of implants after crestal maxillary sinus floor elevation. Measurement and analysis were conducted at six postoperative timepoints (0 months, 6 months, 12 months, 18 months, 24 months, and 30 months) using cone beam computed tomography (CBCT) data on 31 patients from the Chenghuaxinguanghua Dental Clinic who underwent crestal maxillary sinus floor elevation, involving 38 graft sites. The materials resorption rates of the bone graft height (BH) and bone graft width (BW) were assessed. BH and BW resorption rates followed the same trend (*p* = 0.07), with BH and BW resorption rates decreasing with time (r_BH_ = −0.32, *p* < 0.01; r_BW_ = −0.18, *p* < 0.01), and were maximal in the 0–6 month interval, with BH and BW resorption rates of 3.42%/mth and 3.03%/mth, respectively. The average monthly BH and BW resorption rates in the 6–12 month interval rapidly decreased to 1.75%/mth and 1.29%/mth, respectively. The monthly BH and BW resorption rates in the 12–30 month intervals stabilized at 1.45%/mth (*p* > 0.05) and 1.22%/mth (*p* > 0.05), respectively. The higher the initial bone graft height (BH_0_), the lower the BH resorption rates (r_BH_ = −0.98, *p* < 0.05), and the BW resorption rate was different for different graft sites (*p* = 0.01). The resorption rates of bone graft materials implanted through crestal maxillary sinus floor elevation decreased rapidly within the first 12 months post operation and remained stable after 12 months. BH_0_ was identified as a significant factor influencing the resorption rates of bone graft materials. These results could suggest dentists should pay attention to the trend of resorption rates over time and carefully manage the initial height of bone grafts and inspire the research of new bone grafting materials for crestal maxillary sinus floor elevation.

## 1. Introduction

In dental implant procedures, addressing insufficient bone volume in the posterior maxilla is often achieved through maxillary sinus floor elevation [1], involving implanting bone graft materials to enhance bone volume. Crestal maxillary sinus floor elevation, particularly the internal approach, offers advantages such as minimal postoperative reactions, reduced intraoperative trauma, and shortened surgical duration. It has become the routine approach for increasing vertical bone volume in the posterior maxilla [2,3]. The stability of bone graft materials volume is a crucial factor influencing the success of implants [4,5]. Extensive research has been conducted on the resorption dynamics of bone graft materials, including parameters such as BH, BW, and volume, following their placement in the maxillary sinus floor. For instance, Jamcoski et al. [6] conducted a 15-year retrospective study on bone graft materials in the maxillary sinus, observing an initial 15% reduction in the volume of xenogeneic bone graft materials in the first few days post implantation, followed by volume maintenance during the bone remodeling process, demonstrating high predictability. Wiltfang et al. [7] investigated bone graft materials implanted through crestal maxillary sinus floor elevation, revealing a resorption value of approximately 20% after 12 months, with minimal subsequent resorption, but Shanbhag et al. [8] observed the continuous remodeling and resorption rates of bone graft materials in the maxillary sinus floor.

Researchers [9,10,11,12,13,14] studied bone graft materials’ ongoing remodeling and resorption processes on the maxillary sinus floor, and they found that factors influencing resorption might include patient’s gender and age, type of bone graft material, type of implant, bone graft baselines that include residual bone height (RBH), BH_0_, initial bone graft width (BW_0_), whether the surgery was staged, the bone graft site in the maxillary sinus elevation, and the morphology of the maxillary sinus floor. To explore the resorption rate characteristics of bone graft materials in crestal maxillary sinus floor elevation, this study set the null hypothesis that the resorption rates is not correlated with factors such as time, bone graft baselines, patient’s age and gender, type of bone graft materials, or bone graft site. The study aimed to identify factors dentists should consider when implanting bone graft materials during the crestal maxillary sinus floor elevation procedure and to inspire researchers to develop bone graft materials specifically for this purpose.

## 2. Materials and Methods

### 2.1. Patient Inclusion and Exclusion

Basic information and postoperative follow-up CBCT images of patients who underwent crestal maxillary sinus floor elevation at Chenghuaxinguanghua Dental Clinic between 2018 and 2020 were collected, with the data collection ending on 5 October 2022. The inclusion criteria for patient data collection were: (1) patients with no age and gender restrictions; (2) no acute or chronic periodontitis, maxillary sinus disease, or systemic illnesses; and (3) complete postoperative clinical data. The exclusion criteria were: (1) uncontrolled periodontitis, maxillary sinus disease, or systemic illnesses; (2) use of medications known to affect bone metabolism; (3) heavy smokers (more than 10 cigarettes per day) or habitual alcohol drinkers; (4) pregnant or breastfeeding women; (5) missing basic information or postoperative CBCT images. This study followed the Declaration of Helsinki (World Medical Association’s Declaration of Helsinki, 2002). This study received approval from the Chenghuaxinguanghua Dental Clinic Institutional Review Board (approval number: CDCIRB-D-2022-201).

### 2.2. Surgical Procedure

All crestal maxillary sinus floor elevation procedures were conducted at the Chenghuaxinguanghua Dental Clinic, and the surgeries were performed by the same surgeon in strict accordance with surgical protocols. The bone graft material used was either biphasic calcium phosphate (BCP, OSTENOII DT7G0205050, Dentium Co., Ltd., Seoul, Republic of Korea) or deproteinized bovine bone mineral (DBBM, Bio-Oss GEI001001-YL, Geistlich Pharma AG, Wolhusen, Lucerne, Switzerland) implanted into the floor of the maxillary sinus through the alveolar ridge. The Dentium implants (Dentium Co., Ltd., Seoul, Republic of Korea) were thread implants with sand-blasting and acid etching surface treatment, connected with morse taper implant platform.

### 2.3. Data Measurements

**Figure 1:**
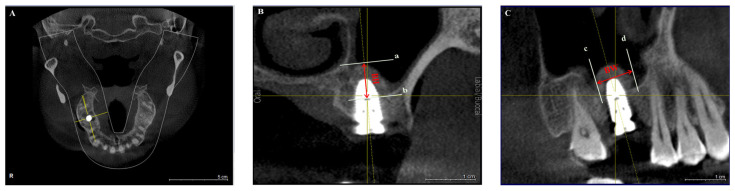
Schematic representation of bone graft materials height and length measurements. (**A**) Horizontal plane. (**B**) Longitudinal plane. (**C**) Transverse plane.

All included CBCT images were acquired using the Sirona 3D Dental Imaging System (Dentsply Sirona Inc.; Charlotte, NC, USA) following a standardized protocol. The acquisition parameters were as follows: field of view of 12 inches (F mode), tube voltage of 85 kVp, tube current of 21 mA/s, and acquisition period of 14 s. The CBCT scans for each patient were transferred to a desktop computer and reformatted using the 3D dental imaging system for analysis in horizontal, longitudinal, and transverse planes. Figure 1 illustrates the linear measurements for BH and BW. The center of each implant was located in the horizontal plane (Figure 1A). In the longitudinal plane, lines a and b were oriented perpendicular to the implant’s central axis, intersecting with the bone graft materials’ top edge and the maxillary sinus’s bottom, respectively. The distance between lines a and b was measured along the implant’s central axis, representing BH (Figure 1B). In the transverse plane, lines c and d were parallel to the implant’s central axis, intersecting with the bone graft materials’ distal and mesial edge. Perpendicular to the implant’s central axis, the distance between lines c and d was measured, representing BW (Figure 1C). The distance between the maxillary sinus floor and the alveolar ridge’s top, known as RBH, is derived from the patient’s preoperative clinical data. BH and BW were measured at six postoperative timepoints: 0 months, 6 months, 12 months, 18 months, 24 months, and 30 months. The same individual took three measurements for each dataset and calculated the averages.

### 2.4. Data Calculation and Analysis

Calculate the resorption rates of BH and BW at x − (x + 6) month intervals and express them as percentages using Equations (1) and (2).

(1) $$\mathrm{Rates}\;\mathrm{of}\;\mathrm{BH}\;\mathrm{resorption}\;\mathrm{at}\;x-(x+6)\;\mathrm{months}\;\mathrm{interval}=\;\frac{{\mathrm{BH}}_{x}-{\mathrm{BH}}_{x+6}}{{\mathrm{BH}}_{x}}\times 100\%$$

(2) $$\mathrm{Rates}\;\mathrm{of}\;\mathrm{BW}\;\mathrm{resorption}\;\mathrm{at}\;x-(x+6)\;\mathrm{months}\;\mathrm{interval}=\;\frac{{\mathrm{BW}}_{x}-{\mathrm{BW}}_{x+6}}{{\mathrm{BW}}_{x}}\times 100\%$$

BH_x_ and BH_x+6_ represent the BH at x months and x + 6 months post operation, respectively, while BW_x_ and BW_x+6_ represent the bone graft width at x months and x + 6 months post operation, respectively.

We analyzed the impact of factors such as postoperative time, bone graft baselines, patient’s age and gender, type of bone graft materials, and bone graft site on the resorption rates of BH and BW.

### 2.5. Statistical Methods

The statistical analysis of the measured data was conducted using SPSS 26 (SPSS Inc., Chicago, IL, USA) and GraphPad Prism 9.5 (GraphPad software LLC., San Diego, CA, USA) was utilized for data visualization. The data for each group are presented as mean ± standard deviation. Normality analysis was performed using the Shapiro–Wilk test. A generalized linear mixed model (LMM) was employed to analyze the primary factors influencing the resorption rates of bone graft materials. The Bonferroni correction test was used to analyze the differences in resorption rates at different timepoints. A significance level of *p* < 0.05 was considered statistically significant.

## 3. Results

### 3.1. Inclusion of Patient’s Cases

A total of 31 patients were included in this study, comprising 38 cases with eligible crestal maxillary sinus floor elevation transplant positions information. Refer to Table 1 and Appendix A for details.

This study categorized age and gender according to the age groups defined by the United Nations World Health Organization [15]. It marked and grouped the grafted sites according to the Palmer notation system [16].

### 3.2. Change in Bone Graft Materials Resorption Rates

**Figure 2:**
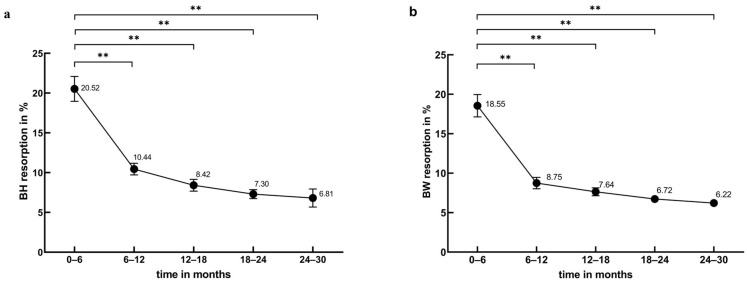
Change in resorption rates of bone graft materials. (**a**) BH resorption in the 0–30 months post operation (** *p* < 0.01). (**b**) BW resorption in the 0–30 months post operation (** *p* < 0.01).

**Figure 3:**
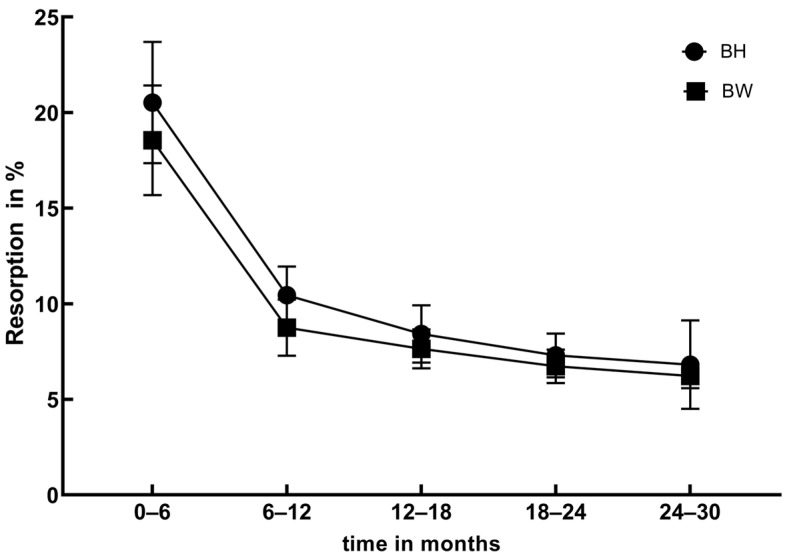
Comparison of BH and BW resorption rates.

The BH and BW measurements obtained at six postoperative timepoints are presented in Appendix B and Appendix C. Statistical analysis was conducted to assess the resorption rates of BH and BW over five postoperative intervals. Figure 2a,b illustrate that within the first 12 months, BH and BW resorption rates decreased significantly (BH: *p* < 0.01, BW: *p* < 0.01). From 12 to 30 months post operation, BH and BW resorption rates remained stable (BH: *p* = 0.18, BW: *p* = 0.34). During the initial six months post operation, BH and BW exhibited the highest resorption rates, with average resorption rates of 20.5 ± 9.64% and 18.55 ± 8.72%, respectively. This translates to average monthly resorption rates of approximately 3.42%/mth for BH and 3.03%/mth for BW. In the 12 months post operation, BH and BW resorption rates rapidly decreased to 10.45 ± 4.54% and 8.75 ± 4.48%, respectively, corresponding to average monthly rates of 1.75%/mth and 1.45%/mth. In the 12–30 months post operation, BH and BW resorption rates stabilized at 7.79 ± 4.38% and 7.33 ± 3.29%, respectively, equivalent to average monthly rates of 1.29%/mth and 1.22%/mth. Statistical analysis revealed a significant correlation between BH and BW resorption rates and time (r_BH_ = −0.32, *p* < 0.01; r_BW_ = −0.18, *p* < 0.01). Figure 3 illustrates the trends in BH and BW resorption rates were similar within the 30 months post operation, with no statistically significant differences (*p* = 0.70).

### 3.3. Factors Influencing Bone Graft Materials Resorption Rates

#### 3.3.1. Bone Graft Baselines

The mean values for BH_0_, BW_0_, and RBH for the 38 cases of crestal maxillary sinus floor elevation transplant positions were 7.53 ± 1.79 mm, 8.45 ± 2.08 mm, and 6.30 ± 1.70 mm, respectively. We analyzed the impact of bone graft baselines on the resorption rates of BH and BW over the 0–30 month interval post operation, and the results are presented in Table 2. BH_0_ had a statistically significant impact on the resorption rates of BH (*p* < 0.01), while RBH and BW_0_ showed no statistically significant impact on the resorption rates of BH and BW (*p* > 0.05)

#### 3.3.2. Age and Gender

**Figure 4:**
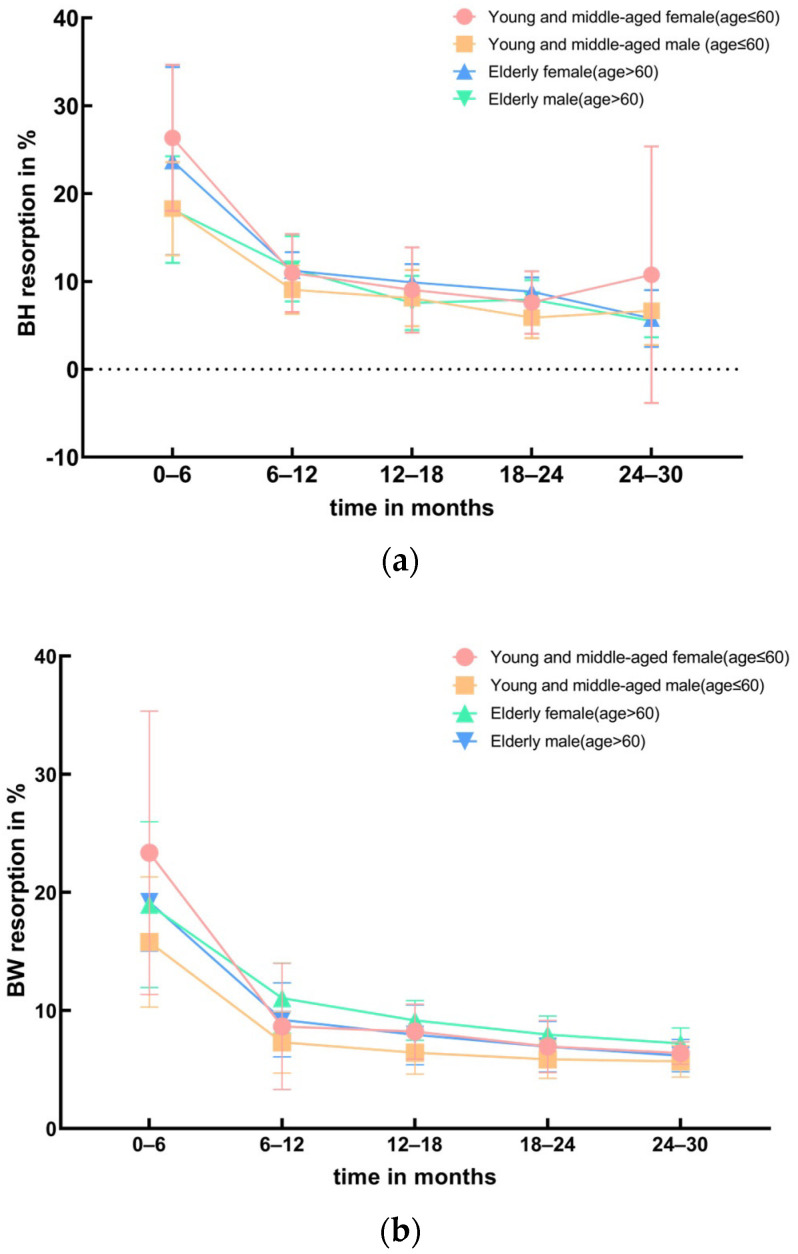
Comparison of BH and BW resorption rates in different ages and genders. (**a**) BH resorption. (**b**) BW resorption.

Figure 4a,b demonstrate that during the 0–30 month interval post operation, the trend differences in the resorption rates of BH and BW over time among 31 patients of different genders and ages are not statistically significant (BH: *p* > 0.05, BW: *p* > 0.05).

#### 3.3.3. Type of Bone Graft Materials

**Figure 5:**
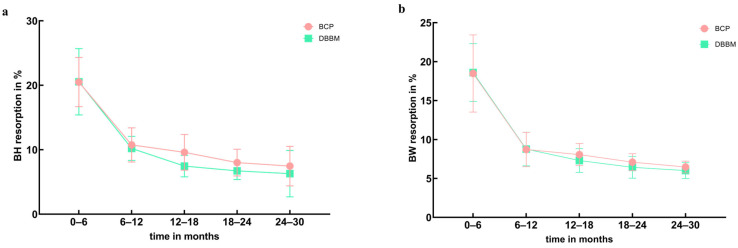
Comparison of BH and BW resorption rates in different types of bone graft materials. (**a**) BH resorption. (**b**) BW resorption.

Figure 5a,b illustrate the trends in BH and BW resorption rates over the 0–30 month interval post operation for different types of bone graft materials. The differences in trends remain statistically insignificant (BH: *p* = 0.73, BW: *p* = 0.26).

#### 3.3.4. Bone Graft Site

**Figure 6:**
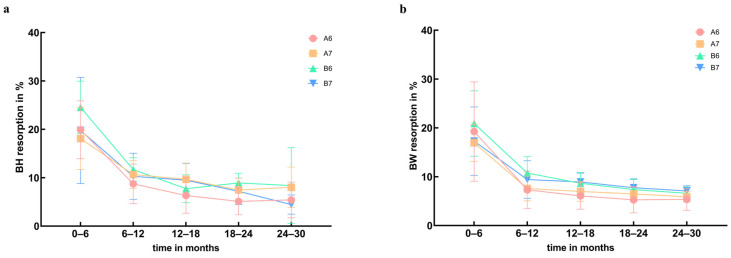
Comparison of BH and BW resorption rates in different bone graft sites. (**a**) BH resorption. (**b**) BW resorption.

Figure 6a,b depict the trends in BH and BW resorption rates over the 0–30 month interval post operation for different bone graft sites. BH resorption rates at different bone graft sites tend to remain statistically insignificant (*p* = 0.06). BW resorption rates at different bone graft sites tend to remain statistically significant (*p* = 0.01).

## 4. Discussion

### 4.1. Characterization of Bone Graft Material Resorption Rates

The long-term stability of bone graft materials after crestal maxillary sinus floor elevation has been a focal point in implant research. The success of implants relies on the adequacy of bone quantity and quality in the implant area and on the long-term stability of bone graft materials. Rios et al. [17] suggested that the change in resorption over time in the bone graft area directly impacts the survival rates of implants. Therefore, a precise understanding of changes in postoperative bone graft material resorption rates and the use of an adequate amount of bone graft materials can enhance the long-term success of implants.

In this study, we analyzed the change in BH and BW resorption rates of bone graft materials implanted into the crestal maxillary sinus floor over a 0–30 month postoperative interval based on actual clinical data. During the healing stage, osteogenic and osteoclastic cells from the blood may enter into the bone graft material framework [18], leading to a faster resorption rate within the first six months post operation as the rates of bone resorption exceed bone generation. As new bone forms and enters the stable phase within the bone graft materials, influenced by factors such as maxillary sinus mucosa and sinus pneumatization [19], the resorption rates of healed bone graft materials rapidly decrease within the first year and remain stable. Similar overall trends were observed in the resorption rates of BH and BW; this suggested that the resorption of bone graft materials is equally influenced in both the vertical and horizontal directions.

Research by Riachi et al. [20] found the vertical resorption rates of bone graft materials volume to be 18.37–21.71%/yr in the first year, decreasing to 3.34%/yr after that. Another study by Moy et al. [21] reported an initial resorption rate of 3.55%/mth within the first two years, followed by an average decrease to 0.58%/mth over the subsequent eight years. In this study, the bone graft materials’ average resorption rates for BH and BW during the initial six months were 3.42%/mth and 3.03%/mth, respectively. The mean resorption percentages during this interval were 20.5 ± 9.64% for BH and 18.55 ± 8.72% for BW. After one year, the average resorption rates decreased to 1.29%/mth for BH and 1.22%/mth for BW, with mean resorption percentages of 7.79 ± 4.38% and 7.33 ± 3.29%, respectively. The observed trend in resorption rates aligns closely with the findings of the two studied above.

### 4.2. Analysis Factors of Resorption Rates

#### 4.2.1. Baselines Analysis

RBH may serve as a source of cells and blood supply during crestal maxillary sinus floor elevation procedures, playing a crucial role in the osteogenesis and mineralization of bone graft materials [22,23]. The literature demonstrated that when the residual bone height in the alveolar ridge is ≥5 mm, the success rates of implants after crestal maxillary sinus floor elevation is significantly high [24]. Some clinical protocols [25,26] suggested opting for lateral window maxillary sinus floor elevation when the remaining bone height is <5 mm to avoid the risk of sinus membrane tearing. In a study on the height variation of bone graft materials in the maxillary sinus, Khijimatgar et al. [27] found that the height resorption rates of bone graft materials in the group with higher RBH were significantly higher than those in the lower RBH group. However, Taschieri et al. [28] studied the impact of RBH on spatial change in bone graft materials after crestal maxillary sinus floor elevation and concluded that RBH did not correlate with the resorption of bone graft materials. The results of this study aligned with the findings of Taschieri; based on the current data analysis, RBH did not influence the resorption rates of bone graft materials in crestal maxillary sinus floor elevation (BH: *p* > 0.05, BW: *p* > 0.05).

The anatomy of the maxillary sinus is complex, and implanting bone graft material too high increases the risk of Schneiderian membrane perforation [4,29]. Therefore, researchers recommend that BH_0_ is limited to 5 mm–9.5 mm [30,31], with the bone graft material covering 2–3 mm above the apex of the implant [32]. The measured average BH_0_ in this study was 7.53 ± 1.79 mm, meeting the recommended BH_0_, with no cases of maxillary sinus membrane perforation observed.

Zheng et al. [33] found that the larger the initial volume of bone graft materials, the greater the tension on the Schneiderian membrane during maxillary sinus elevation. This tension may be converted into pressure on the bone graft materials, leading to a change in its spatial morphology. Kuo et al. [30] conducted a study using BCP for crestal maxillary sinus floor elevation and observed that the higher the BH_0_, the greater the resorption rates of bone graft materials. However, this study found that the higher BH_0_ was associated with slower resorption rates of bone graft materials (*p* < 0.01). The analysis suggested that the higher BH_0_ increases the contact area with the Schneiderian membrane, promoting the entry of more mesenchymal stem cells and blood into the bone graft materials, accelerating its osteogenesis and mineralization [34,35], thereby reducing the resorption rates of bone graft materials.

#### 4.2.2. Age and Gender Analysis

The impact of human hormones and blood supply on bone formation is crucial, and these factors are collectively influenced by age and gender. Sex hormones, including estrogen (such as estradiol) and testosterone, affect bone metabolism and growth processes by influencing osteoblast cells. Oxygen, nutrients, and growth factors in the blood are essential for supporting bone cell activity and repair [36]. Consequently, it is observed that elderly individuals, especially older women, are more susceptible to osteoporosis, resulting in significant bone resorption [37]. However, researchers such as Arora et al. [38] contend that gender and age did not impact the resorption of bone graft materials. According to the data analysis in this study, we did not find any differences in the resorption rates of bone graft materials among the 31 patients of different genders and ages (BH: *p* > 0.05, BW: *p* > 0.05).

#### 4.2.3. Type of Bone Graft Materials Analysis

DBBM, BCP, and other bone graft materials exhibit excellent biocompatibility. The inter-material interstices provide blood and osteocyte adhesion channels, endowing them with osteoconductive properties. These materials are widely used in maxillary sinus floor elevation procedures [39,40,41]. A comparative study by Tomas et al. [35] investigated the histological differences between DBBM and BCP as bone graft materials following maxillary sinus lift procedures. The study found no statistically significant differences in spatial change between the two types of bone graft materials postoperatively. According to the current data analysis in this study, no significant differences had been observed in the resorption rates of bone graft materials based on the different types of bone graft materials (BH: *p* > 0.05; BW: *p* > 0.05).

#### 4.2.4. Bone Graft Site Analysis

Mildly increased air pressure within the maxillary sinus cavity is usually associated with respiration, and the elevated pressure can lead to bone resorption and the re-pneumatization of the maxillary sinus after crestal maxillary sinus floor elevation. The sinus ostium is located on the medial wall of the maxillary sinus, and the airflow through the open ostium causes the movement of the maxillary sinus mucosa. Different bone graft sites in the sinus are subjected to varying continuous air pressures, ultimately influencing the healing and structure of the bone graft materials [42]. Franceschetti et al. [43] found no statistically significant differences in bone graft materials resorption among different tooth positions. Consistent with the current analysis, their study did not identify any differences in bone graft materials BH resorption rates among different bone graft sites (*p* > 0.05) or any differences in bone graft materials BW resorption rates among different bone graft sites (*p* < 0.05).

## 5. Conclusions

To ensure the long-term success of implants in the posterior maxillary region, a clear understanding and control of the long-term stability and resorption rate change of the bone graft materials surrounding the implants are essential. This study observed that the resorption rates of bone graft materials implanted through crestal maxillary sinus floor elevation vary over time: they rapidly decreased within the first year and remained stable afterward. The height of the initial bone graft materials was a crucial factor influencing resorption rates, with higher initial bone graft heights associated with lower resorption rates. Bone graft sites affect the bone graft width resorption rates.

This study recommends that dentists pay attention to the changing trends in the resorption rates of bone graft materials over time and aim to implant the maximum possible BH_0_ within a total elevation range of 5 mm to 9.5 mm, which would help to reduce the re-sorption rate of bone graft material in the maxillary sinus floor and maintain the space for new bone growth. This study also suggests that material researchers should develop bone graft materials for crestal maxillary sinus floor elevation procedures to allow dentists to implant as much BH_0_ as possible at the maxillary sinus floor. Due to limitations in follow-up time and case numbers, future studies could include more data for analyzing the long-term resorption rates of bone graft materials and influencing factors like particle size, implant model, and type of implant platform.

## Figures and Tables

**Table 1:** Statistical overview of basic information for 38 cases of crestal maxillary sinus floor elevation.

Group	Category	Quantity
Age and Gender (Pts)	Young and middle-aged female (≤60 years)	6
	Young and middle-aged male (≤60 years)	12
	Elderly female (>60 years)	5
	Elderly male (>60 years)	8
Type of bone graft materials (cases)	BCP	17
	DBBM	21
Bone graft site (cases)	A6	8
	A7	12
	B6	10
	B7	8

Note: A/B6: right/left upper first molar; A/B7: right/left upper second molar.

**Table 2:** Analysis of the effects of bone graft baselines on BH and BW resorption rates.

Resorption Rate	Statistical Indicator	RBH	BH_0_	BW_0_
BH	r	−0.20	−0.98	0.17
	*p*	0.41	<0.01	0.36
BW	r	0.04	−0.22	−0.17
	*p*	0.79	0.14	0.13

## Data Availability

The original contributions presented in the study are included in Appendix A, Appendix B and Appendix C, further inquiries can be directed to the corresponding author.

## Appendix A

**Table A1:** Data of the basic information of 31 patients with crestal maxillary sinus floor elevation.

Patient	Gender	Age	Bone Graft Site	RBH (mm)	Type of Bone Graft Material
1	Male	55	A6, A7	7.57	BCP
2	Female	55	B6	6.43	BCP
3	Male	79	B6	4.59	BCP
4	Male	84	B6, B7	5.1	BCP
5	Male	82	A7	7.53	BCP
6	Female	72	A7	4.17	BCP
7	Male	63	A7	3.17	BCP
8	Male	48	B7	8	BCP
9	Male	48	A7	6.34	BCP
10	Male	35	B7	6.06	BCP
11	Male	72	A6, B6	7.41	DBBM
12	Male	56	A7	7.19	DBBM
13	Male	46	A6	6.08	DBBM
14	Male	55	A7	8.02	DBBM
15	Male	64	A7	9.57	DBBM
16	Male	44	A7	5.88	DBBM
17	Male	51	A6	5.95	DBBM
18	Female	47	B7	5.24	DBBM
19	Male	48	B7	6.51	DBBM
20	Female	54	B6	8.25	DBBM
21	Female	65	B6	6.5	DBBM
22	Male	54	A6, A7	5.53	DBBM
23	Female	56	A6	5.59	BCP
24	Male	37	A6	9.4	DBBM
25	Female	75	B6, B7	8.46	DBBM
26	Male	71	B6	7.86	DBBM
27	Male	67	B6, B7	3.73	DBBM
28	Female	52	B6	6.21	DBBM
29	Female	63	A6, A7	3.77	BCP
30	Female	57	B7	7.79	BCP
31	Female	73	A7	3.95	BCP

## Appendix B

**Table A2:** Data of BH (mm) in the 38 cases of the crestal maxillary sinus floor elevation in the 0–30 month intervals post operation.

Number	Patient	Graft Site	Time (Months Post Operation)					
			0	6	12	18	24	30
1	1	A6	9.59	8.36	7.8	9.18	8.24	6
2	1	A7	7.59	5.47	4.94	7.29	7.02	6.8
3	2	B6	6.28	4.5	3.97	6.23	6.21	6.18
4	3	B6	9.94	7.6	7.05	5.04	4.99	4.88
5	4	B6	5.41	3.94	3.36	7	6.85	6.1
6	4	B7	5.37	4.3	3.32	2.87	2.6	2.34
7	5	A7	6.26	4.58	4.08	3.8	3.53	3.41
8	6	A7	8.9	6.62	6.01	5.36	5.03	4.82
9	7	A7	9.85	8.27	8.11	8.01	7.48	7.07
10	8	B7	7.72	6.97	6.64	6.18	5.77	5.38
11	9	A7	7.2	5.23	4.19	8.92	8.78	8.47
12	10	B7	7.43	6.49	5.8	4.94	4.68	4.54
13	11	A6	6.19	4.76	4.13	8.34	7.03	6.86
14	11	B6	8.95	6.42	6.06	6.68	6.42	6.17
15	12	A7	9.36	7.75	6.79	4.66	4.04	3.88
16	13	A6	8.67	7.91	7.71	5.77	5.4	5.21
17	14	A7	4.52	4.19	3.86	5.2	4.88	4.81
18	15	A7	4.53	4.37	3.68	6.59	5.55	5.36
19	16	A7	10.55	9.21	8.38	5.04	4.67	4.46
20	17	A6	10.77	8.9	8.4	4.41	4.17	4.02
21	18	B7	8.89	6.3	5.93	6.94	6.93	6.68
22	19	B7	9.33	8.19	7.31	6.59	6.28	6.09
23	20	B6	6.68	4.42	3.71	7.7	7.56	7.37
24	21	B6	6.11	3.98	3.43	4.92	4.7	4.6
25	22	A6	8.8	6.39	6.28	3.29	2.86	2.66
26	22	A7	10.66	6.61	5.77	3.39	3.17	3.09
27	23	A6	7.11	6.31	5.65	5.4	4.75	4.03
28	24	A6	8.21	6.2	5.42	3	2.68	2.51
29	25	B6	4.99	4.25	3.77	4.38	4.15	3.96
30	25	B7	8.44	4.91	4.48	7.52	7.19	6.97
31	26	B6	5.64	4.75	4.25	9.27	9.02	8.92
32	27	B6	7.65	6.61	5.9	4.05	3.72	3.58
33	27	B7	6.4	6.26	5.49	6.01	5.54	5.46
34	28	B6	8.08	6.05	5.14	7.05	6.71	6.42
35	29	A6	6.04	4.89	4.2	4.44	3.98	3.72
36	29	A7	5.15	4.04	3.67	4.2	3.76	3.6
37	30	B7	6.21	4.32	4.04	5.06	4.74	4.67
38	31	A7	6.72	6.17	5.41	4.51	4.23	4.12

## Appendix C

**Table A3:** Data of BW (mm) in 38 cases of crestal maxillary sinus floor elevation in the 0–30 month intervals post operation.

Number	Patient	Graft Site	Time (Months Postoperatively)					
			0	6	12	18	24	30
1	1	A6	11.8	8.79	8.63	8.01	7.48	7.07
2	1	A7	11.04	10.24	9.67	9.18	8.61	7.99
3	2	B6	6.23	3.89	3.55	3.28	3.07	2.88
4	3	B6	10.72	8.24	7.21	6.44	5.92	5.53
5	4	B6	10.55	8.77	7.71	6.95	6.35	6.09
6	4	B7	14.16	10.9	9.71	8.87	8.19	7.6
7	5	A7	9.24	7.88	6.87	6.2	5.68	5.3
8	6	A7	11.38	8.43	7.59	6.89	6.49	6.17
9	7	A7	10.82	9.33	8.97	8.92	8.78	8.47
10	8	B7	6.69	6.24	5.98	5.72	5.49	5.19
11	9	A7	7.92	6.58	5.75	5.17	4.69	4.27
12	10	B7	9.33	8.93	7.65	6.8	6.14	5.62
13	11	A6	10.66	9.45	8.7	8.32	8.01	7.6
14	11	B6	9.44	7.86	7.75	7.54	7.41	7.04
15	12	A7	10.4	8.93	8.55	8.34	7.86	7.44
16	13	A6	9.89	6.95	6.54	6.23	6.02	5.99
17	14	A7	9.22	7.43	6.96	6.68	6.42	6.17
18	15	A7	6.67	5.21	5.07	4.66	4.32	4.15
19	16	A7	8.68	7.57	6.74	6.18	5.72	5.35
20	17	A6	5.84	5.14	5.1	5.04	4.99	4.88
21	18	B7	8.02	6.24	5.48	4.9	4.44	4.09
22	19	B7	9.09	7.98	7.24	6.69	6.37	6.03
23	20	B6	8.74	7.24	6.17	5.65	5.25	4.98
24	21	B6	5.24	3.89	3.3	3	2.74	2.57
25	22	A6	7.58	7.31	7.15	6.86	6.71	6.31
26	22	A7	8.27	6.49	5.76	5.2	4.78	4.42
27	23	A6	6.26	4.28	3.83	3.48	3.24	3.02
28	24	A6	5.7	3.7	3.23	2.91	2.63	2.45
29	25	B6	7.22	5.74	5.05	4.49	4.08	3.73
30	25	B7	7.2	5.75	5.02	4.51	4.09	3.74
31	26	B6	8.07	5.45	4.58	3.98	3.52	3.16
32	27	B6	5.53	4.77	4.29	3.87	3.52	3.24
33	27	B7	6.8	5.22	4.65	4.2	3.84	3.55
34	28	B6	9.05	8.59	8.18	7.84	7.58	7.12
35	29	A6	8.19	6.27	5.46	4.91	4.46	4.09
36	29	A7	7.61	6.84	6.21	5.65	5.18	4.79
37	30	B7	6.35	4.67	4.63	4.2	3.84	3.6
38	31	A7	5.45	5.08	4.81	4.55	4.31	4.04
